# Evaluation of the diagnostic capacity for neglected tropical diseases in a refugee settlement in Uganda: a cross-sectional study

**DOI:** 10.1186/s41182-026-00968-w

**Published:** 2026-05-27

**Authors:** Elvis Tamale, Paddy Derrick Malinga, Mable Ssebaduka Nabweteme, Celine Ahurira, Conrad Makai, Kenneth John Ssembuze, Patricia Ajeru Aketoko, Patience Atuhaire

**Affiliations:** 1 University of Cambridge, Cambridge, UK; 2 Faculty of Medicine, Mbarara University of Science and Technology, Mbarara, Uganda; 3 Medical Department, Medical Teams International, Portland, USA; 4 Faculty of Biomedical Sciences, Kampala International University, Kampala, Uganda; 5 Makerere University Johns Hopkins University Research Collaboration, Kampala, Uganda; 6 Department of Internal Medicine, St Mary’s Hospital Lacor, Gulu, Uganda

**Keywords:** Neglected tropical diseases, Refugee settlement, Uganda, Primary healthcare, Health workers, Knowledge, Capacity, Schistosomiasis, Soil-transmitted helminths

## Abstract

**Background:**

Neglected tropical diseases (NTDs) pose a substantial global public health challenge, particularly in vulnerable refugee populations. In Uganda, approximately 1.8 million refugees residing in settlements nationwide, including over 145,000 in Nakivale alone, are at risk, yet primary healthcare (PHC) facility capacity within refugee settlements to effectively manage these diseases remains underexplored. This study assessed infrastructure, diagnostic capabilities, and resource availability for NTD management in health facilities across Nakivale Refugee Settlement, Uganda.

**Methods:**

A quantitative assessment was conducted across 8 health facilities (6 Health Centre III, 2 Health Centre IV) in Nakivale Refugee Settlement. Data collection included facility type, bed capacity, dedicated laboratory services and personnel, availability of diagnostic tests and guiding tools, status of reporting systems, and essential medicines and laboratory supplies for soil-transmitted helminths (STH) and schistosomiasis. Descriptive statistics summarized findings.

**Results:**

The 8 assessed facilities (6 HCIII, 2 HCIV) averaged 23 and 50 beds, respectively. All facilities reported dedicated laboratories with technicians and microscopes. Diagnostic tests (microscopes, dyes, assay strips) and guiding tools (clinical guidelines, laboratory protocols, bench aids) were consistently available, though specific test types varied. Five out of eight (62.5%) facilities had Health Management Information System reporting forms. Essential medicines, albendazole and mebendazole, were available in most facilities (7 had both, 1 had albendazole only). Common laboratory supplies (e.g., centrifuge tubes, syringes) were universal, but critical items like cellophane and Kato–Katz templates were absent in all facilities. Most facilities were over 10 km from the town council, the service hub.

**Conclusion:**

Health facilities in Nakivale Refugee Settlement possess foundational infrastructure for NTD management, including laboratories, microscopes, and essential medicines. However, significant gaps exist in specific diagnostic supplies (e.g., Kato–Katz templates, cellophane) and reporting system completeness. Their predominantly remote location also highlights potential access challenges. Addressing these gaps through targeted provision of laboratory consumables, strengthening reporting, and considering geographical accessibility is crucial for enhancing PHC capacity to manage NTDs in this refugee setting.

**Supplementary Information:**

The online version contains supplementary material available at 10.1186/s41182-026-00968-w.

## Introduction

Neglected tropical diseases (NTDs) are a group of 13 infections caused by parasitic worms, protozoa, or bacteria currently affecting over 1 billion people, with another 1 billion at risk of infection [1–14]. They strike the world’s poorest people with adverse effects on the health, well-being, and socio-economic facets [2, 15–18]. Africa bears a substantial portion of this global burden, accounting for over 600 million cases, particularly affecting poor rural communities [1, 2, 19, 20]. Uganda is endemic for all five NTDs, with more than 40 million people at risk for one or more NTDs targeted by the United States Agency for International Development (USAID) Act to End NTDs [21]. Despite their significant burden, NTDs are largely preventable and amenable to elimination. In 2020, the World Health Organization (WHO) outlined a roadmap for eradicating and eliminating 20 NTDs by 2030 [22, 23]. The WHO, alongside policymakers and academics, advocates for integrating NTD management into existing public health programs within endemic countries [5, 24]. Such integration is posited to contribute significantly to achieving several sustainable development goals (SDGs), particularly those related to water, sanitation, and poverty reduction, as being essential for the sustainable elimination of NTDs [14, 22, 25, 26]. Despite the burden of NTDs in sub-Saharan Africa, NTDs have not been well integrated into the PHC of most countries. Previous studies in Nigeria, Tanzania, and Burundi have consistently reported low capacity and readiness among health workers to diagnose and manage NTDs [1, 27–29]. The Ministry of Health in Uganda has identified several hindrances to eliminating NTDs by 2030, including a lack of knowledge, skills, and equipment for diagnosis and management in health facilities and deficiencies in reporting systems [30]. Uganda currently hosts approximately 1.99 million refugees, the largest population in Africa, with over 92% residing in 13 designated rural settlements across 12 districts. These settlements are primarily concentrated in West Nile region (hosting refugees from South Sudan) and the Southwestern region (hosting those from the DRC, Rwanda, and Burundi). Nakivale Refugee Settlement, situated in Isingiro District of Southwest Uganda, is the eighth-largest settlement globally, hosts approximately 145,613 refugees from ten different nationalities [18, 31]. Within this settlement, NTDs, particularly schistosomiasis and soil-transmitted helminths (STHs), are prevalent, with reported rates of 26.6% and 26.5%, respectively [30]. The increasing number of refugees, coupled with inadequate resources, raises critical questions about the capacity of the existing health system to effectively diagnose, manage, and treat these NTDs. Moreover, comprehensive data on NTDs and their prevalence are scarce in regions such as southwestern Uganda. Uganda’s national strategy for NTDs involves integrated control programmes [32]. This study assessed the capacity of primary healthcare centers to timely diagnose, treat, and manage soil-transmitted helminths and schistosomiasis among refugees in Nakivale Refugee Settlement, Southwestern Uganda, by looking at the availability of essential diagnostic and reporting infrastructure.

## Methods

### Study design and setting

This study employed a quantitative, cross-sectional design focusing on assessing health facility capacity. The study was conducted in Nakivale Refugee Settlement, located in southwestern Uganda, a large settlement hosting a diverse refugee population.

### Study population and sampling

The study population comprised 8 health facilities within Nakivale Refugee Settlement. These facilities included 6 Health Centre III (HCIII) and 2 Health Centre IV (HCIV) facilities, representing key points of primary healthcare service delivery in the settlement. At the time of the study, Nakivale Refugee Settlement was served by approximately 10 public health facilities. Of these, eight facilities comprising six Health Centre III (HC III) and two Health Centre IV (HC IV) were purposively selected for inclusion. This selection was based on their status as the primary diagnostic hubs within the settlement, as these tiers are the only ones equipped with the laboratory infrastructure and specialized personnel required for the management and surveillance of neglected tropical diseases.

### Data collection

Data were collected by trained research assistants using a structured quantitative assessment form, adapted from WHO health facility modules. The tool underwent expert review for content validity and was piloted at a comparable Health Centre III in Isingiro District; based on the pilot, technical laboratory terms were refined to ensure consistent interpretation. The assessment was conducted over one month from February to March 2025 to accommodate the staggered availability of Facility In-Charges and laboratory personnel across the settlement. At each of the eight facilities, a single, comprehensive assessment was performed. Data were triangulated from three primary sources: (1) a retrospective review of laboratory and pharmacy registers; (2) a physical audit of equipment and administrative inventories; and (3) structured interviews with lead technical staff. This cross-referencing ensured a high degree of data accuracy regarding NTD diagnostic and management capacity. The assessment tool utilized is available as Supplemental Material 1.

The form captured facility characteristics (type, location, and bed capacity), laboratory capacity (availability of a dedicated lab, technician, and microscope), and diagnostic resources (tests for intestinal schistosomiasis and STH, alongside clinical guidelines). Reporting infrastructure was assessed through the Health Management Information System (HMIS) and the availability of reporting forms. The survey also documented essential medicines (praziquantel, albendazole, mebendazole, and ivermectin) and laboratory supplies, including slides, centrifuges, filters, and Kato–Katz templates.

Written informed consent was obtained from all participants, including Facility In-Charges (for administrative access) and purposively selected Laboratory Technicians and Clinicians. Participants were briefed on the study’s objectives, the voluntary nature of their participation, and the confidentiality of their data before signing.

### Data analysis

Completed questionnaires were first logged into Microsoft Excel 2016 for data entry, cleaning, and coding to ensure accuracy and consistency, after which the dataset was exported to STATA 17.0 for analysis. Facility characteristics such as facility type and distance from the town council were analyzed as categorical variables and summarized using frequencies and percentages, while the number of hospital beds was treated as a continuous variable and summarized using means and ranges. Laboratory capacity (presence of a laboratory, technician, and microscope), diagnostic resources (availability of tests and diagnostic guidelines), reporting infrastructure (HMIS use and reporting forms), essential medicines, and laboratory supplies were all analyzed as binary categorical variables (present = 1, absent = 0) and summarized descriptively using frequencies and proportions. Multi-select categories, such as diagnostic tests and laboratory consumables, were further disaggregated to present the availability of each item across the eight facilities, allowing a detailed profile of service readiness.

### Ethics statement

This study was conducted in accordance with the ethical principles outlined in the Declaration of Helsinki. Ethics approval was obtained from the Lacor Hospital Institutional Research Ethics Committee (Reference Number: LACOR-2024-362). Written informed consent was obtained from all participants prior to data collection. Participants were informed about the study objectives, procedures, potential risks and benefits, their right to decline or withdraw at any time without any consequence, and confidentiality measures were assured.

## Results

### Baseline characteristics of health facilities

The study assessed a total of eight health facilities within the Nakivale Refugee Settlement to determine their foundational capacity for NTD management. All eligible health facilities within Nakivale Refugee Settlement that met the inclusion criteria were assessed. Therefore, the study represented a census of facilities within the settlement rather than a sampled subset. The baseline institutional and geographical characteristics of these facilities are presented in Table 1. Of these, 6 were identified as Health Centre III (HCIII) facilities, and 2 were Health Centre IV (HCIV) facilities. Regarding geographical accessibility, 7 out of the 8 facilities were located more than 10 km from the town council, with only one Health Centre IV situated within 10 km. The average number of hospital beds varied by facility type. Health Centre III facilities reported an average of 23 hospital beds, while Health Centre IV facilities had a higher average of 50 hospital beds. All 8 assessed health facilities reported the presence of a dedicated laboratory and a designated laboratory technician for the diagnosis of Neglected Tropical Diseases. All facilities (100%) were equipped with functional binocular electric microscopes. This foundational infrastructure allowed for the consistent performance of microscopic examinations.

**Table 1:** Baseline characteristics of assessed health facilities (*N* = 8)

Characteristic	Category	Summary	
		Count	Percentage (%)
Total facilities assessed		8	N/A
Facility type	Health Center III	6	75
	Health Center IV	2	25
Geographical accessibility	> 10 km from town council	7	87.5
	Within 10 km from town council	1	12.5
Average hospital bed capacity	Health Center III	23 beds	N/A
	Health Center IV	50 beds	N/A
Laboratory presence	Dedicated Laboratory	8	100
	Designated Lab Technician	8	100
	Microscope availability	8	100

Values are counts and percentages; N/A indicates not applicable

### Availability of diagnostic tests and guiding tools

The assessment evaluated the availability of specific commodities and diagnostic aids required for the management of soil-transmitted helminths (STH) and schistosomiasis. Table 2 details the inventory of essential medicines, laboratory supplies, and clinical guidelines found at the sites. Seven out of the 8 facilities reported having both albendazole and mebendazole available. One facility reported having only albendazole. All facilities, however, lacked praziquantel and ivermectin.

**Table 2:** Critical gaps in essential NTD diagnostics and treatment medications

Characteristic	Item	Availability	
		Facilities with item	Percentage (%)
Availability of essential medicines	Albendazole/mebendazole (both)	7	87.5
	Albendazole only	1	12.5
	Praziquantel	0	0
	Ivermectin	0	0
Availability of key lab supplies	Centrifuge tubes	8	100
	Syringes	8	100
	Cellophane	0	N/A
	Kato–Katz templates	0	N/A
	Urine dipsticks	7	87.5
	Dyes	5	62.5
	Spatula	3	37.5
	Membrane filters	2	25
	Iodine	4	50
	Malachite green	1	12.5
	Glycerine solution	1	12.5
Available diagnostic tests for intestinal schistosomiasis and STH	Clinical guidelines	7	87.5
	Laboratory protocols	6	75
	Bench aids	5	62.5

Values are counts and percentages; N/A indicates not applicable

The availability of specific diagnostic tests for intestinal schistosomiasis and STH showed some variation across facilities, but commonly included microscopes, dyes, and assay strips. In terms of tools to guide diagnosis, all facilities reported the presence of at least some of the listed items, such as clinical guidelines, laboratory protocols, and bench aids, though the specific combination of these tools differed between facilities.

### Reporting systems

The status of the Health Management Information System (HMIS) was examined to determine the consistency of data collection for NTD diagnosis and treatment. While 5 of the 8 facilities had reporting forms available for the Health Management Information System, 3 facilities reported that these forms were not available. The availability of standardized reporting forms is summarized in Table 3.

**Table 3:** Inconsistent NTD reporting infrastructure and geographical accessibility challenges

Characteristic	Category	Summary	
		Facilities	Percentage (%)
Availability of reporting forms	Reporting forms for HMIS are available	5	62.5
	Reporting forms for HMIS not available	3	37.5

Values are counts and percentages

## Discussion

This study identified four major gaps in the readiness of health facilities in Nakivale Refugee Settlement to provide comprehensive NTD services, which included inadequate diagnostic supplies despite the presence of basic laboratory infrastructure, absence of essential medicines such as praziquantel and ivermectin, weak reporting systems, and geographical inaccessibility of most facilities. These limitations constrain the effectiveness of NTD prevention, diagnosis, and management, despite some encouraging strengths such as universal availability of laboratories, technicians, and microscopes.

Whereas all the facilities assessed reported having a designated laboratory technician and a functional microscope, critical diagnostic supplies were either inconsistently available or completely absent. When compared to other studies done in similar remote settings, the stronger baseline diagnostic infrastructure was an improvement, unlike similar studies in Tanzania and Burundi, where laboratory capacity was far more limited [33, 34]. However, although centrifuge tubes and syringes were available in all facilities, essential tools for WHO-recommended procedures such as urine sedimentation, centrifugation, and the Kato–Katz method (e.g., Kato–Katz templates, cellophane, and malachite green) were unavailable. While routine stool examinations in Uganda primarily utilize direct wet mounts for broad parasite detection, this study employed the Kato–Katz technique, the WHO-recommended standard for epidemiological surveys, to specifically quantify the prevalence and intensity of *Schistosoma mansoni* and soil-transmitted helminths within this high-risk refugee population. This pattern mirrors findings from Tanzania, where facilities relied heavily on urine dipsticks and direct smear microscopy due to similar supply gaps [33]. While urine dipsticks and direct smears are useful for rapid screening, their low sensitivity undermines accurate disease burden estimation, especially in high-risk refugee populations.

There was a noted absence of praziquantel and ivermectin across all facilities, despite their inclusion in the WHO Model List of Essential Medicines for schistosomiasis and onchocerciasis control [29]. In contrast, albendazole and mebendazole were widely available (87.5%), reflecting an imbalance in NTD drug procurement. This finding is particularly concerning given the high prevalence of schistosomiasis in refugee-hosting regions of Uganda [35]. Similar stock-outs have been documented in other low-resource settings, where fragmented supply chains and donor-driven drug distribution lead to inconsistent medicine availability [36]. Without praziquantel and ivermectin, facilities in Nakivale Refugee Settlement remain unable to deliver integrated, guideline-based NTD care.

There were inconsistencies with the reporting infrastructure, with only 62.5% of facilities having HMIS reporting forms. This inconsistency limits systematic data capture, surveillance, and accountability. Similar reporting gaps have been observed in other sub-Saharan African health systems, where underreporting of NTD cases undermines programmatic planning and resource allocation [37]. Strengthening reporting mechanisms is particularly critical in refugee settlements, where disease dynamics differ from national averages due to overcrowding, poor sanitation, and frequent mobility.

With regard to facility location, 87.5% of facilities were located more than 10 km from the town council, with only one HCIV within 10 km. Many settlement inhabitants stay near the town councils, and this distance barrier adds to the structural challenges refugees face in accessing care. Previous studies have consistently highlighted that increased distance to health facilities reduces care-seeking behavior, increases delays, and contributes to poor adherence to(38) long-term treatment regimens [38]. For NTDs that require periodic preventive chemotherapy, such barriers undermine program reach and effectiveness.

This study’s strengths include its ability to provide a detailed, baseline assessment of diagnostic and treatment readiness within a major African refugee settlement, a setting often overlooked in national health surveys. By triangulating data from facility registers, physical inventories, and staff interviews, the study highlights specific bottlenecks that are immediately actionable for policymakers.

The study is limited in that it focused on a single refugee settlement which limits the generalizability of the findings to other humanitarian contexts. Additionally, while the study confirmed the availability of hardware, it did not evaluate the quality of diagnostic practices, the consistency of water and power utilities, or the efficacy of waste management. Furthermore, the lack of data on external supportive supervision and quality assurance means the functional accuracy of the reported diagnostics remains unverified.

## Conclusion

In conclusion, this assessment shows that while Nakivale Refugee Settlement’s health facilities have a solid foundation for NTD management with laboratories, microscopes, and essential medicines in place, critical gaps remain. The lack of specific diagnostic supplies like Kato–Katz templates and cellophane, gaps in reporting systems, and the remote location of most facilities all pose significant challenges to effective NTD control. To address these, a regular supply of key laboratory consumables should be ensured through quarterly procurement reviews and better stock management. Strengthening and digitizing NTD reporting systems is also essential to improve surveillance and planning. Practical solutions to overcome remoteness, such as mobile outreach clinics or community-based diagnosis, should be prioritized.

Finally, future research should utilize qualitative methods to investigate effect of distance, the socio-economic barriers to service use and the systemic challenges staff face in maintaining laboratory resources. Such insights are vital to tailoring interventions and accelerating NTD elimination in this vulnerable setting.

## Supplementary Information

Supplementary material 1.

## Data Availability

The datasets generated and/or analyzed during the current study are not publicly available due to the sensitive nature of participant data and to maintain participant anonymity, but are available from the corresponding author on reasonable request.

## Abbreviations

NTDs: Neglected tropical diseases
PHC: Primary healthcare
WHO: World Health Organization
HCIII: Health Centre III
HCIV: Health Centre IV
STH: Soil-transmitted helminths
SDG: Sustainable Development Goal
USAID: United States Agency for International Development
