# Supplementary material for: Evaluation of the diagnostic capacity for neglected tropical diseases in a refugee settlement in Uganda: a cross-sectional study
Source: Trop Med Health. 2026 May 27;54:97. doi: 10.1186/s41182-026-00968-w (PMC13214166; doi:10.1186/s41182-026-00968-w)
Supplement: Supplementary file 1 — Supplementary material 1. [file 41182_2026_968_MOESM1_ESM.pdf]

Capacity and readiness of healthcare facilities to diagnose and manage Neglected Tropical Diseases in Eastern Uganda

Quantitative questionnaire for health workers

1. Age (in years)
2. Gender (Male or Female)
3. Type of Health Facility working at (Health Centre 4, Health Centre 3, District hospital)
4. Education level of health worker (Degree, Certificate, Diploma)
5. Position held at health facility (according to MoH guidelines)
6. Knowledge of NTDs (ask the participant to list examples)
7. Any previous training on NTDs (soil-transmitted helminths, schistosomiasis)
    a. If any, the duration

Knowledge

1. Knowledge of S. haematobium symptoms: knowledge of symptoms of S. haematobium, Haematuria, Oliguria, Dysuria, Polyuria, Nephrotic colic, Anaemia, Asthenia [Yes/No]
2. Knowledge on S. mansoni symptoms: Diarrhoea, bloody stool, abdominal pain, hepatosplenomegaly, hematemesis, anaemia, asthenia [Yes/No]
3. Knowledge on the presentation of STH (hookworm, Ascaris, whipworm): diarrhoea, abdominal pain, chronic malnutrition, reduced immunity, anaemia, cough
4. Knowledge on medications used in the management of STH and schistosomiasis
    a. Praziquantel for schistosomiasis
    b. Albendazole or mebendazole for A. lumbricoides, T. Trichuris and hookworms
    c. Ivermectin for S. stercoralis

Reporting practices

1. Any previous training on disease surveillance reporting
    a. If any, duration
2. Ever been visited for supportive supervision for NTDs [Yes/No]
3. Ever received a penalty for not reporting [Yes/No]
4. Requires monetary incentive to report on NCDs [Yes/No]
5. Find reporting form easy to fill in [Yes/No]

Quantitative assessment for each Health facility

1. Type of health facility (Health Centre 4, Health Centre 3, District hospital)
2. Distance of health facility from town council
3. Number of hospital beds
4. Availability of dedicated lab and lab technician for diagnosis of NTDs
5. Availability of diagnostic tests for intestinal schistosomiasis and STH
6. Availability of clinical guidelines, laboratory protocols or bench aids to guide diagnosis

7. Reporting of diagnosis and treatment to the Health Management Information System
    a. Availability of reporting forms at health facility
8. Availability of essential medicines for management of STH and schistosomiasis
9. **Supplementary table:** Assessment of available laboratory equipment in the health facilities offering laboratory services

Equipment		%
**Microscope**		
Yes		
No		
**Object and cover slides**		
Yes		
No		
**Centrifuge**		
Yes		
No		
**Membrane filters**		
Yes		
No		
**Filter holders**		
Yes		
No		
**Syringes**		
Yes		
No		
**Centrifuge tubes**		
Yes		
No		
**Iodine (5%)**		
Yes		
No		
**Urine dipstick**		
Yes		
No		
**Malachite green**		
Yes		
No		
**Methylene blue**		
Yes		
No		
**Glycerine solution 50%**		
Yes		

No		
**Cellophane**		
Yes		
No		
**Kato Katz template**		
Yes		
No		
**Spatula**		
Yes		
No		

Qualitative interview guide

Instructions:

- The interview should be conducted in a quiet setting that the health worker is familiar with. The interview should be conducted by one member of the research team assisted by a note taker who will take notes during the discussion.
- An audio recorder should be used to record the proceedings.
- Before commencing discussion, health workers need to voluntarily offer written consent
- Before commencing the discussion, ensure to fill out the socio-demographic form
- Information obtained from these interviews will help us understand the capacity and readiness of health facilities in Eastern Uganda to manage NTDs.

Discussion questions

1. Tell me about the burden of NTDs in this area
   a. *Probe the commonest NTDS (Soil-transmitted helminths, schistosomiasis) and their causes. Guide them if they're not sure what NTDs are.*
   b. *Probe on why they think the burden (or severity) is high or low (sanitation and excreta management, community awareness, access to safe water, zoonotic reservoirs)*
   c. *Probe on an approximate number of cases seen in the last month and year.*
   d. *Probe on age group that is most burdened (pregnant women for schistosomiasis, school going children for STH)*
   e. *Probe on number of deaths, and burden on the community in terms of productivity*
2. Tell me about how the common NTDs are transmitted
   a. *Specifically, probe about the transmission of soil-transmitted helminths and schistosomiasis*
   b. *Probe on specific behaviours that confer transmission that may be common in these communities (defecation in water bodies, open defecation, rice farming, swimming in water bodies, walking bare foot)*
3. Tell me about how NTDs are managed in this area
   a. *Probe on what the common clinical presentation is for schistosomiasis and the different STH (Haematuria, Oliguria, Dysuria, Polyuria, Nephrotic colic, Anaemia, fatigue)*
   b. *Probe on what people usually do when they get the above-mentioned signs and symptoms (traditional care, report to health facilities, self-treatment)*
   c. *Probe on any hindrances to people presenting to health facilities (such as stigmatization, lack of community awareness, knowledge on STH and schistosomiasis in the communities)*
   d. *Probe on how diagnosis is made at the health facility (point of care diagnostics, resistance testing)*
   e. *Probe on what other conditions may be mistaken for STH or schistosomiasis*
   f. *Probe on community screening and community sensitization*
   g. *Probe on common complications seen*

   h. *Probe on treatments used (that is for the different STH and schistosomiasis*
   i. *Probe on use of preventive chemotherapy (for schistosomiasis that is mass drug administration, albendazole for school going children)*

4. Tell me about the challenges do you face in the management of NTDs?
   a. *Probe on health system challenges: drug stock outs, shortage of personnel, lack or shortage of clinical guidelines, lack of motivation of health workers that handle these conditions, lack of knowledge among health workers*
   b. *Probe on availability of diagnostic tests at all PHC facilities (point of care diagnostics,*
   c. *Probe on drug resistance (i.e., re-occurrence of schistosomiasis even when treated)*
   d. *Probe on case surveillance and reporting to the HMIS*
5. Tell me about what has been done to tackle these challenges
   a. *Probe on Continuous Medical Trainings, community sensitization,*
6. Can you suggest any solutions to the challenges you mentioned?
7. Is there anything else the interviewee would like to share with the interviewer?
